# Using respondent-driven sampling (RDS) to recruit illegal poly-substance users in Cape Town, South Africa: implications and future directions

**DOI:** 10.1186/s13011-016-0074-1

**Published:** 2016-09-01

**Authors:** Nadine Harker Burnhams, Ria Laubscher, Simon Howell, Mark Shaw, Jodilee Erasmus, Loraine Townsend

**Affiliations:** 1 Alcohol, Tobacco and Other Drug Research Unit, South African Medical Research Council, P.O. Box 19070, Tygerberg, 7505 Cape Town, South Africa; 2 School of Public Health and Family Medicine, Falmouth Building, Faculty of Health Sciences University of Cape Town, 7701 Cape Town, South Africa; 3 Biostatistics Unit, South African Medical Research Council, P.O. Box 19070, Tygerberg, Cape Town, 7505 South Africa; 4 Centre for Criminology, University of Cape Town Rondebosch, Private Bag x3, Cape Town, 7701 South Africa; 5 Centre of Criminology, Private Bag x3, Cape Town, 7701 South Africa; 6 Health Systems Research Unit, South African Medical Research Council, P.O. Box 19070, Tygerberg, Cape Town, 7505 South Africa

**Keywords:** Poly-substances, Respondent-driven sampling, South Africa, Illicit Drugs

## Abstract

**Background:**

South Africa continues to witness an increase in illicit poly-substance use, although a precise measurement continues to be compounded by difficulties in accessing users. In a pilot attempt to use respondent-driven sampling (RDS)—a chain referral sampling method used to access populations of individuals who are ‘hard-to-reach’—this article documents the feasibility of the method as recorded in a simultaneously run, multisite, poly-substance study in Cape Town. Here we aim to a) document the piloting of RDS among poly-substance users in the three socio-economic disparate communities targeted; b) briefly document the results; and c) review the utility of RDS as a research tool.

**Methods:**

Three cross-sectional surveys using standard RDS procedures were used to recruit active poly-substance users and were concurrently deployed in three sites. Formative research was initially conducted to assess the feasibility of the survey. To determine whether RDS could be used to successfully recruit poly-substance users, social network characteristics, such as network size was determined.

**Results:**

A 42.5 % coupon return rate was recorded in total from 12 initial seeds. There were vast differences in the recruitment chains of individual seeds—two generated more than 90 recruits, and 2 of the 10 recruitment chains showing a length of more than 10 waves. Findings include evidence of the use of 3 or more substances in all three sites, high levels of unemployment among users, with more than a third of participants in two sites reporting arrest for drug use in the past 12 months.

**Conclusions:**

Our results indicate that RDS was a feasible and acceptable sampling method for recruiting participants who may not otherwise be accessible. Future studies can use RDS to recruit such cohorts, and the method could form part of broader efforts to document vulnerable populations.

## Background

Over the last two decades, South Africa has witnessed an increase in the use of illegal substances and number of users—many of whom use more than one substance, termed poly-substance abuse [1]. While such increases have been mirrored both globally and in the country, poly-substance use remains particularly high in the Western Cape province, with some 44 % of those patients accessing specialist alcohol and drug treatment services reporting the use of multiple drugs [2]. Routine data collected to assess treatment demand by the South African Community Epidemiology Network on Drug Use (SACENDU) has furthermore found that patients who primarily use methamphetamine also report using cannabis and methaqualone [3] as secondary substances. Moreover, many of those patients who have reported heroin as their primary substance often also use methamphetamine as a secondary substance [2].

**Fig. 1:**
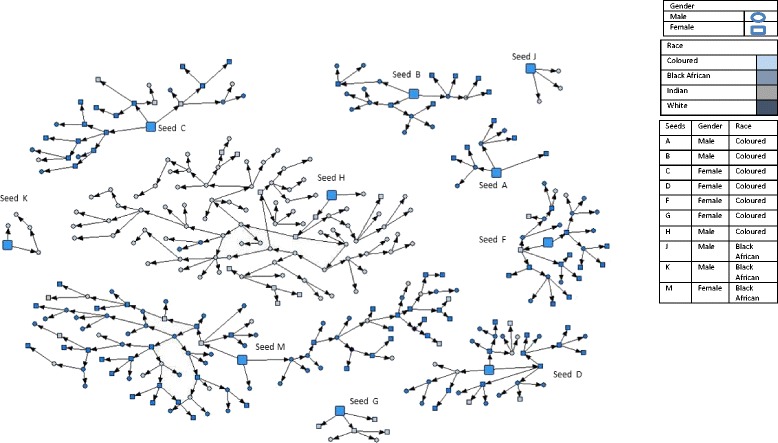
Recruitment Network Diagramme

While these data are obtained from specialist substance abuse treatment services and only provide information on those that are able to access treatment [4] rather than being indicative of trends in the general population, they point to a burgeoning problem. Indeed, little is known about the wider use of multiple illegal substances, especially in cohorts who are unable to access specialist substance abuse treatment services. Obtaining data from sources other than treatment demand trends, is important considering its significance as a source of information on local, regional and national patterns of substance use, and is integral in effectively delineating resources and the planning of treatment and prevention services for poly-substance using populations. From the perspective of governance, data remain central in decreasing the burden of harm associated with poly-substance use. Additionally, the use of multiple illegal substances magnify the health risks associated with substance use [5], since they increase the likelihood of the emergence of severe psychological problems, and impact on a plethora of social concerns both directly and indirectly. For instance, poly-substance use has been found to lead to both intended and unintended injuries, death and disability as well as multiple health problems including but not limited to cardiovascular disease, diabetes and the acquisition of infectious diseases such as tuberculosis and the Human Immunodeficiency Virus (HIV). Risks for the development of mood and other behaviour disorders and executive functioning impairments following consumption of illicit substances are also heightened [6]. The identification and positioning of poly-substance users in targeted research therefore remains critical to public health in South Africa [7].

Despite this, recruiting representative samples of illicit poly-substance users who are not already registered as in-treatment patients remains an on-going challenge in substance abuse research [8]. While there are multiple reasons for this, one of the most salient is that individuals who engage in activities that are illegal or stigmatised are often hesitant to participate in research, as they may be fearful of the possible legal repercussions or of further social stigmatisation [9]. Recruitment strategies that rely on users’ relationships, rather than operating as externally-based interventions, therefore become an important means of engaging with this cohort. One such strategy, respondent-driven sampling (RDS), is an increasingly-used chain referral sampling method that has previously shown utility in accessing populations of individuals who are ‘hard-to-reach’ [10].

RDS relies on respondents themselves to recruit people of interest from the target population, founded on the convenience sampling of selected initial respondents (‘seeds’) drawn from the intended cohort. Once a seed is found eligible for participation—as a function of meeting the predetermined criteria and on completion of the study procedures—they are compensated for participation (the ‘primary incentive’) but also further provided with a set number of coupons (usually 2 to 3) with which to recruit others who meet the eligibility criteria from their social networks [11]. This process is stimulated by providing seeds with a ‘secondary incentive’ for recruits who successfully complete the study procedures. Enrolled participants then in turn serve as recruiters, and are offered the same primary and secondary incentives. This procedure creates an expanding system of chain referrals characterized by ‘waves’ of recruitment, which continue until the desired sample size is reached [11, 12].

RDS has been utilised extensively among marginalised and hard to reach populations in both local and international HIV research [7, 8, 13–15]. Moreover, injecting drug users (both as illicit substance users and as a population most at risk of acquiring and transmitting HIV) have also been the subject of HIV biological and behavioural surveillance research in other settings [9, 12, 15, 16]. While extensive research employing RDS methodologies have been used to successfully reach injecting drug using populations [17–22], few RDS-driven studies among illegal poly-substance users have been conducted in South Africa, with only one known previous study among methamphetamine users in the Western Cape Province [7]. Given that relatively fewer RDS studies have been conducted among poly-substance users, Daniulaityte and colleagues stresses the importance of formative work prior to embarking on an RDS study. For instance, in their study of non-dependent pharmaceutical opioid users, RDS recruitment was less efficient than expected (due to narrow eligibility criteria), and several modifications had to be made to make RDS work within this target population [23]. The authors therefore highlight the importance of determining whether the target population is socially connected enough (given eligibility criteria) to generate a “representative” sample using RDS methodology [21].

As such, the purpose of this article is to, a) document the piloting of RDS among poly-substance users in three socio-economically and racially disparate communities in Cape Town; b) to briefly document the levels of poly-substance use found in the sample; and c) to review the primary demographic and other variations revealed by the use of the method.

## Methods

From 26 January to 24 February 2015 we conducted three cross-sectional surveys using respondent-driven sampling (RDS) among poly-substance users residing in three suburbs in Cape Town, South Africa. To our knowledge, the simultaneous deployment of RDS aimed at poly-substance users, across multiple sites, had not been attempted before in South Africa.

### Formative research

Prior to the pilot survey, we conducted formative research to assess the appropriateness and feasibility of using RDS to collect survey data from the study population. The questions used in the formative Focus Group Discussions (FGDs) were based on those recommended beforehand by Johnston and colleagues [12]. During these FGDs, participants were asked to comment on their social networks, their perceived acceptability of RDS, appropriate venues, type and value of incentives, and were also used to identify potential seeds. Six FGD (three with males and three with females) were conducted in the three study settings. The division by sex was employed in an attempt to mitigate the stigma associated with substance abuse, particularly for women. A summation of the information from the FGDs ascertained that there is an underlying social network of poly-substance users in each of the study settings; i.e. poly-substance users knew each other and were socially networked. Participants were confident that members of their social networks would complete the survey procedures, which included a survey and the on-site testing of urine samples to confirm poly-substance use. We were able to establish an appropriate type and value for incentives, acceptable venues, and study operation times. Many FGD participants were willing to act as initial recruiters (seeds) to begin the recruitment process.

### Setting

As noted, the study was conducted in three communities in the Cape Town metropolitan area chosen for their economic, cultural and ethnographic diversity. Site 1 has an estimated population of 156 000 where the median monthly income is R1601 (approximately US$133). Site 2, on the outskirts of the Cape Town central business district, has an estimated population of 9300 people, and the monthly median income is R18801 (approximately US$1566). Site 3 is a ‘township’ approximately 15 kilometres east of Cape Town. It has an estimated population of 392 000. The median monthly income in this community is R1301 (approximately US$108). Reliable population data is difficult to obtain, however the last national census (conducted in 2011) reported that the three sites had different racial makeups, as tabulated below (Table 1):

The survey was conducted in three centrally located, easily accessible permanent venues in each of these study sites, and all had private bathrooms for collection and testing of urine samples. One-on-one eligibility screening and the completion of the computer-based survey by interviewers was done in large halls where participants were seated at tables that were discreetly separated from each other to maximize privacy. In total, the study employed nine fieldworkers, or three per site, who also conducted the urine sample analyses as part of eligibility screening and for which they had been specially trained. Weekly feedback and debriefing sessions were held with all fieldworkers and were facilitated by the principal- and co-investigators. Prior to this, all study personnel underwent a week-long training regime that included research ethics, respondent-driven sampling procedures, field operation procedures and familiarization with the electronic barcoding system, and the computer-based questionnaire.

### Procedures

Participants were eligible if they were 18 years or older; living, working or socializing in each of the study settings; and had used two or more of six illicit substances (confirmed by the urine analysis) – cocaine, cannabis, methamphetamine, amphetamine, opiates, and methaqualone.

Eleven ‘seeds’ (See Fig. 1) were initially recruited and enrolled in the study during the first week of operation: four seeds in sites one and two, and three in the third site. An additional seed was recruited in one site at the beginning of the third week of operation. The same eligibility criteria were applied to seeds as study participants. Each seed and recruit was provided with three recruitment coupons with which to facilitate the recruitment of eligible people into the survey. Participants were provided with detailed instructions by the specially trained fieldworkers on how to engage those who met the eligibility criteria, incentivised with supermarket shopping vouchers to the value of R60 (approximately US$5), passed on at completion of the survey and on provision of the urine sample. Additional shopping vouchers to the value of R20 (approximately US$1.67) were given to participants for each recruit who successfully completed the survey. Recruitment coupons had unique, barcoded numbers that were used to link recruiters to their recruits. We used barcode numbering software, specially developed for our purposes, to generate the barcoded recruitment coupons (bryantresearchsystems.com).

In Site 1 we reduced the number of recruitment coupons to zero during week two of operation because the site had been overwhelmed with respondents wishing to participate. At this time, 47 respondents had participated. The seed recruited later in this site received three coupons until the last two days of operation, when coupons were reduced to zero in anticipation of the closure of this site. In total 190 coupons were issued at this site, and at study closure, 137 recruits had participated (a return rate of 66.3 %). In Site 2 we reduced the number of recruitment coupons to two on the second last day of operation, and zero on the final day. In total 283 coupons were issued at this site, and at study closure, 111 recruits had participated (a return rate of 36.4 %). In Site 3, we reduced the number of coupons to two on day 12 of operation, to one on day 15, and to zero on final day. In total 320 coupons were issued at this site, and at study closure, 126 recruits had participated (a return rate of 37.5 %).

We implemented RDS using standard procedures [12]. Once it was established that they met the eligibility criteria, had provided informed written consent, and passed the urine screener, they went on to complete the computer-based survey. Data were collected by means of a computer-based survey questionnaire (CBSQ) composed of 185 questions. In maximising our limited interactions, we asked questions that measured socio-demographic information; substance use patterns and frequencies, quantities and prices of drugs; alcohol use; risk behaviours such as unprotected and transactional sex, criminal activity; and screened for symptoms of drug dependence using CAGE (Cutting down, Annoyance, Guilt feelings, Eye-opener) - Questions Adapted to Include Drug Use CAGE-AID [25]. To further prevent or prohibit repeat participation, the fieldworkers who were on site every day were trained to be on the lookout for repeaters. If a repeater was suspected, s/he would be confirmed by other fieldworkers and, on consensus, be told to leave. All study activities were conducted in the language of the participant’s choosing (Afrikaans, isiXhosa or English).

### Measures

#### Social network size

Analysis of data from RDS surveys requires an accurate social network size from each participant as the data is weighted using this information. We used four questions to maximize the accurate reporting of each participant’s personal social network size:

- Question #1: How many people do you know, who know your name and you know theirs, and you have seen them in the past 30 days?
- Question #2: How many of the people from question #1 are 18 years or older?
- Question #3: How many of the people from question #2 have used drugs in the past 30 days?
- Question #4: How many of the people from question #3 have used more than one drug in the past 30 days?

The response to this final question was used as the number representative of each participant’s social network size.

#### Demographic items

The CBSQ included a section providing information on age, gender, and employment and education level. Questions regarding participants’ socio-economic status (SES) using the Family Affluence Scale (FAS) [24] were also included.

#### Poly-substance use

The use of illegal substances was assessed by means of urine analysis and alcohol use was self-reported in the past 7 days. Use of any of these substances (excluding alcohol) was scored as 1 and each participant was given a poly-substance use score by summing the scores. We dichotomized the poly substance score to identify those who had used two to three substances, and those who had used more than three substances during a maximum of the past 7 days.

#### Risk behaviours

Question items related to trading sex for drugs, theft and arrests in the last 12 months were taken from a questionnaire used in the 3-metro study on drugs and crime in South Africa [3]. Responses to questions were: 1 = ‘Yes’; 2 = ‘No’ and 3 = ‘Refuse to Answer’.

#### CAGE

A self-report four-item test with questions on Cutting down, Annoyance at criticism, Guilty feelings and use of an Eye-opener was also used to screen for symptoms of a substance-related disorder. On the CAGE [25] two or more positive replies suggest symptoms for a substance-related disorder.

#### Treatment

To determine whether participants had ever received substance abuse treatment, are currently undergoing treatment or intending to receive treatment, three single questions were inserted into the questionnaire. Participants were asked to give a 1 = ‘Yes’; 2 = ‘No’ and 3 = ‘Refuse to Answer’ response to the three questions.

### Sample size

To assess the feasibility of using RDS, the current study, which was a pilot study sought to recruit a minimum of 100 poly-substance users in each community.

### Data analysis

Sample proportions, estimates of population proportions and 95 % confidence intervals (CIs) were calculated using the freely-available Respondent-Driven Sampling Analysis Tool 7.1.38 (RDSAT) (www.respondentdrivensampling.org) for poly-substance use and demographics. Using information about each participant’s social network size (degree) and cross- and within-group recruitment patterns (who recruited who, essentially), the RDS I estimator was used to derive population estimates. Given that the network range was 1 –600 with extremely small and large outliers, we chose to pull in 5 % of outliers in the reported network sizes. RDS methodology recommends that a value of 5 % be pulled in the top and bottom of the network size values (RDSAT 7.1. Manual) [26]. We used chi-square tests of association to examine significant differences between the three sites on key variables.

There were very few instances of missing data because the CBSQ did not allow non-responses to questions. All questions included a ‘refuse to answer’ response option. Ethical clearance was obtained from the research ethics committee in the Faculty of Law at the University of Cape Town.

## Results

Over a period of 18 week days (i.e. Monday to Thursday from 10 am to ±4 pm) we issued a total of 793 recruitment coupons of which 374 were redeemed (42.5 %). Coupon return rates and sample sizes for each site are described above. In site 1, 11 people were ineligible as they did not test positive for two or more illicit substances; in site 2, eight people were ineligible —six did not test positive for two or more illicit drugs and two were younger than 18 years; and in site 3, 16 did not test positive for two or more illicit drugs and one was younger than 18 years. Twelve seeds were recruited during the 4½ weeks of operation (5 in site 1, 3 in site 2 and 4 in site 3). Two seeds proved inactive (one in site 2 and one in site 3; two seeds generated more than 90 recruits (one in site 2 and one in site 3); and four seeds generated 20 or more recruits in site 3 (Table 2). In relation to average network size, in Site 1 mean network size was 58.41 (SD = 82.58); Site 2 the mean average was 26.28 with a standard deviation of (17.95), and in Site 3 the average mean network size reported was 87.63 (SD = 154.91).

**Table 1:** Site race profiles (National Census Data): Comparison of proportion of poly-substance users with census data – by site

	Black	White	Coloured	Indian	Other
Site 1					
Population^a^	46.22 %	0.12 %	51.49 %	0.34 %	0.12 %
Poly-substance users recruited	(12.7 %)	(0.8 %)	(86.5 %)	(0 %)	(0 %)
Site 2					
Population^a^	23.26 %	60.95 %	10.85 %	2.20 %	2.76 %
Poly-substance users recruited	(19.4 %)	(3.9 %)	(74.8 %)	(0 %)	(2 %)
Site 3					
Population^a^	98.62 %	0.08 %	0.59 %	0.07 %	0.63 %
Poly-substance users recruited	(98.1 %)	(0 %)	(1.9 %)	(0 %)	(0 %)

^a^National Census Data 2011

**Table 2:** Recruitment patterns depicting number of waves, number of recruits and seed network size in the 3 sites

Seed no	Site	Date enrolled	Waves	Number of recruits	Network size
A	1	26-Jan-15	2	9	85
B	1	26-Jan-15	4	22	10
C	1	11-Feb-15	4	27	30
D	1	26-Jan-15	4	35	7
F	1	27-Jan-15	5	28	20
G	2	27-Jan-15	2	6	22
H	2	27-Jan-15	10	94	4
I	2	27-Jan-15	0	0	21
J	3	28-Jan-15	1	3	60
K	3	28-Jan-15	3	4	45
L	3	28-Jan-15	0	0	50
M	3	28-Jan-15	10	97	6

Two of the 10 recruitment chains had a length of more than 10 waves: in site 2 one recruitment chain produced 84.7 % (94 of 111) of recruits, and in site 3, one recruitment chain produced 76.9 % of recruits (97 of 126). In all sites the majority of respondents (93 %) were recruited by ‘friends’ whom they had seen ‘many times’ during the past 30 days.

### Illicit substance and alcohol use

Across all three sites crystal methamphetamine, cannabis and methaqualone were reported as the dominant substances of use (Table 3). The majority of poly substance users in all sites reported alcohol use in the past 7 days, with more reporting alcohol use in site 3 compared to the other sites. The use of cocaine was more common in Site 1 compared to the other sites; cannabis and methaqualone use was more common in site 3; and the use of opiates, such as heroin, were more common in site 2.

### Demographics

Our results show some variations across sites on key demographic variables (Table 1). Compared to national population estimates for site 1 (46.2 %) the proportion of Black African persons recruited in site 1 was 12.7 %. Similarly, 3.9 % of white participants were recruited in Site 2 with population estimates for Site 2 reported at 60.9 % (Table 1). The proportion of females varied across sites with only 10 % of participants in site 3 identifying as female (Table 4). Across sites, the majority of participants reported not having enough food or would only be able to afford staple provisions (Table 4), while more than half of our sample across two sites (site 1 (87.3 %) and site 2 (61.7 %)) were living in shacks, temporary dwelling or were homeless. Despite participants having high school and/or tertiary education (across sites), unemployment was close to 75 % in site 1, 45.2 % in in site 2 and 80.6 % in site 3.

**Table 3:** Reported use of substances and alcohol in the three study sites^ab^

	Total participants in study (*N =* 337)	No. Participants Site 1 (*N =* 126)	No. Participants Site 2 (*N =* 103)	No. Participants Site 3 (*N =* 108)
	N (%)^c^	N (%)	N (%)	N (%)
Cocaine	36 (10.7 %)	28 (22.2 %)	8 (7.5 %)	1 (1.0 %)
Cannabis	264 (78.4 %)	93 (74.4 %)	70 (65.4 %)	101 (97.1 %)
Methamphetamine/amphetamine	306 (90.9 %)	118 (93.7 %)	95 (88.8 %)	94 (90.4 %)
Opiates	71 (21.0 %)	28 (22.2 %)	41 (38.3 %)	2 (1.9 %)
Methaqualone	254 (75.4 %)	85 (67.5 %)	73 (68.2 %)	96 (92.3 %)
Alcohol	290 (5.9 %)	112 (9.6 %)	82 (76.6 %)	95 (91.3 %)

^a^multiple response item, therefore does not total 100

^b^sample proportions are reported

^c^numerator (percentages) are reported

**Table 4:** Level of poly-substance use, socio-demographic characteristics, risk behaviours, among poly- substance users residing in three communities in Cape Town, South Africa, 2015

	Site 1			Site 2			Site 3			
Variable	Sample N (%)	Adjusted^a^ %	95 % CI	Sample N (%)	Adjusted^a^ %	95 % CI	Sample N (%)	Adjusted^a^ %	95 % CI	x^2^
										(*p-*value)
Poly substance use										
2 –3 substances	45 (35.7)	43.7	23.3 –58.5	53 (51.5)	56.5	44.1 –67.0	24 (22.2)	27.7	15.7 –39.3	
> 3 substances	81 (64.3)	56.3	41.5 –76.7	50 (48.5)	43.5	32.7 –55.9	84 (77.8)	72.3	60.7 –84.3	
Ses variables										
Age										
18 –24	24 (19.0)	35	16.3 –47.6	14 (13.6)	13.5	6.20 –20.7	51 (47.2)	54	38.6 –68.9	53.079
25 –30	43 (34.1)	33.6	23.1 –53.3	31 (30.1)	33.7	23.5 –46.2	35 (32.4)	30.5	19.7 –44.1	0.00
31 –35	29 (23.0)	14.4	6.4 –24.0	17 (16.5)	15.4	6.8 –24.1	13 (12.0)	5.9	0.2 –10.3	
> 35	30 (23.8)	17	7.1 –27.3	41 (39.8)	37.4	26.0 –49.9	9 (8.3)	9.6	2.4 –17.6	
	(mean 31.1; SD 8.3)			(mean age 34.1: SD 8.7)						
Gender										
Male	62 (49.2)	34.8	22.0 –51.8	58 (56.3)	52.7	38.9 –65.1	96 (88.9)	89.6	80.6 –97.6	43.798
Female	64 (50.8)	65.2	48.2 –78.0	45 (43.7)	47.3	34.9 –61.1	12 (11.1)	10.4	2.4 –19.4	0.00
Employment										
Unemployed	95 (75.4)	75.3	61.7 –91.0	47 (45.6)	45.2	34.2 –57.1	87 (80.6)	79.4	68.6 –89.2	34.649
Employed	31 (24.6)	24.7	9.0 –38.3	56 (54.4)	54.8	42.9 –65.8	21 (19.4)	20.6	10.8 –31.4	0.00
Education										
Primary/none	24 (19.0)	16.4	7.9 –27.5	34 (33.0)	34.1	23.9 –46.0	14 (13.0)	17.3	8.1 –28.5	13.254
High/tertiary	102 (81.0)	83.6	72.5 –92.1	69 (67.0)	65.9	54.0 –76.1	94 (87.0)	82.7	71.5 –91.9	–0.001
Ses										
Not enough for food	50 (40.3)	42.6	25.2 –56.6	46 (47.4)	43.5	28.6 –58.7	50 47.2)	43.1	31.8 –56.2	13.378
Basics only	57 (46.6)	50.5	35.9 –68.1	28 (28.9)	33.8	19.6 –49.2	34 (32.1)	37.5	25.7 –50.3	–0.037
Food, clothes + extras	17 (13.7)	6.9	3.2 –12.4	23 (23.7)	22.7	12.3 –35.4	22 (20.8)	19.4	0.1 –27.8	
Risk behaviours										
Had sex for drugs	19 (15.1)	11.6	4.8 –20.5	21 (20.4)	19.4	10.3 –29.6	7 (6.5)	10.5	3.4 –18.9	8.71
										–0.013
Stole to buy drugs	51 (40.5)	34	22.6 –51.8	23 (22.3)	19.7	10.8 –31.8	35 (32.4)	23.3	15.5 –32.9	8.528
										–0.014
Arrested	34 (27.0)	19.4	10.1 –31.3	46 (44.7)	44	32.8 –52.5	43 (39.8)	34.9	25.7 –48.4	8.394
										–0.015
Cage										
Low risk	33 (26.2)	30	13.7 –39.4	25 (24.3)	27.5	16.4 –38.2	19 (17.6)	15.2	8.6 –23.5	2.609
Problematic use	93 (73.8)	70	60.6 –86.3	78 (75.7)	72.5	61.8 –83.6	89 (82.4)	84.8	76.5 –91.4	–0.271
Treatment										
Ever Treatment (No/Yes)	102 (81.0)	75.9	57.9 –89.5	68 (66.0)	63.8	51.8 –76.1	97 (89.8)	88.5	78.0 –95.2	20.992
	24 (19.0)	24.1	10.5 –42.1	35 (34.0)	36.2	23.9 –48.2	11 (10.2)	11.5	4.8 –22.0	0
Future Treatment (No/Yes)	60 (39.7)	38.6	23.6 –54.6	29 (28.2)	32.1	22.2 –43.7	27 (25.0)	26.2	17.2 –37.4	6.563
	76 (60.3)	61.4	45.4 –76.4	74 (71.8)	67.9	56.3 –77.8	81 (75.0)	73.8	62.6 –82.8	–0.038

^a^Weighted estimates

### Rates of poly-substance use

Table 4 above shows that the simultaneous use of 3 or more substances was prevalent in all three sites (56 % in site 1, 44 % in site 2, and 72 % in site 3). Except for site 2, the use of 3 or more substances was higher than the use of 2 or 3 substances, with this difference reaching significance in site 3. Very few people (4 in site 1 and 11 in site 2) had injected an illegal drug.

### Risk behaviours

With respect to engaging in risk behaviours, 11.6 % in site 1, 19.4 % in site 2 and 10.5 % of participants in site 3 had exchanged sex for drugs, while 34 % in site 1, 19.7 % in site 2, and 23.3 % in site 3 had stolen items and goods for drugs. Across two sites (Sites 2 and 3) more than a third of participants reported having been arrested for drugs in the past 12 months (Table 4).

### Problematic poly-substance use and treatment

CAGE self-report showed that 70 % of participates in site 1, 72.5 % in site 2 and 84.8 % in site 3 reported a CAGE score of ≥ 2, indicating symptoms of a substance-related disorder. The majority of participants across all sites were significantly more likely to report that they had never received treatment for a substance-related disorder (75.9 % in site 1; 63.8 % in site 2; and 88.5 % in site 3). While the overall majority of participants indicated no past treatment, more than half of the participants in all three sites reported that they felt they would gain from specialist substance abuse treatment (Table 4).

## Discussion

Our results from this pilot study indicate that RDS was a feasible and acceptable sampling method for recruiting poly-substance users in three socio-economically and racially disparate communities in Cape Town. While coupon return rates were lower in site 2 and 3 when compared to site 1, our coupon return rate in Site 1 matched that of a study conducted by Kimani and colleagues [7]. It can however be argued that had the study sites remained open, we may have had a higher percentage of coupon return rates, but this was a pilot study which only had a target of 100 participants per site, after which sites were closed. Despite this, recruitment occurred rapidly in all three sites suggesting that RDS has the potential to be a cost-effective and efficient means of recruiting larger samples. Similar to a study conducted in Papua New Guinea (a developing country context where RDS methods were employed), our recruitment success could be attributed to the comprehensive manner in which the formative phase, prior to implementation, was conducted since assessing whether the RDS recruitment strategy fits the sub-population grouping is essential [21, 27]. Literature on utilising RDS methods strongly recommend that the formative phase be characterised by careful planning which should involve careful selection of the study sites, in the case of this study, the three areas to ensure easy accessibility to the study sites. The use of trained fieldworkers is also encouraged, and for this study we recruited fieldworkers who were not only well trained but also familiar with the three areas, with some being resident in the areas [28]. The study also employed a field coordinator who was able to attend to immediate needs at sites and Fridays were reserved for intensive debriefing, troubleshooting, reconciling of vouchers and coupons as well as planning for the following weeks work. According to Okal and colleagues, dealing constructively with such practicalities that emanate from the field can improve the theoretical fit of RDS.

While sampling procedures should be capable of reaching all members of the population or subpopulation under surveillance in order to produce unbiased estimates of trends in poly-substance use [28], it is evident from this study that using RDS methodology can have certain limitations. For instance, while the intention was to recruit persons from varying race, gender and socio-economic groupings, it was found that the bulk of poly-substance users recruited via this method were largely homeless persons. Literature suggests that this bias could be linked to the double incentive system utilised by RDS, which may attract mostly indigent persons but it is also likely that the incentive was not of sufficient value to encourage participation of relatively “wealthier” people. While attempts were made to obtain seeds of a higher economic status, we were never able to obtain informed consent, and some reasons cited anecdotally for non-participation were concerns around confidentiality and anonymity, scepticism of the research process and a general lack of interest.

Additionally, we found some demographic differences across sites. Few female poly-substance users were recruited, particularly in site 3. For many South African women, it is still considered taboo to use illicit substances, alcohol or even tobacco, which might explain the low representation of female illegal substance users in this study [7]. Substance use often remains hidden due to the stigma associated with women who use substances [29]. Whatever the reasons for low female participation, efforts to engage female poly-substance users in research, treatment and interventions is vital given that women tend to have more severe substance use disorders, despite having used less of the substance over a shorter period of time compared to men [30, 31]. Women are also more likely to experience health problems such as accelerated courses of liver disease [32], increased risk for premenopausal osteoporosis [33] and fertility problems and breast cancer [34]. Additionally, few people older than 35 years were recruited in site 3. This finding might be explained by culturally-prescribed interaction between younger and older people being regulated and discouraged. It was further found that in sites 1 and 2, RDS was unable to recruit a representative sample as far as race profiles pertain. This could partially be attributable to the lingering legacy of apartheid associated to the fact that those of white heritage generally fall within a higher socio-economic bracket. It is however clear from this study that future research should explore whether these gender, race and age differences are related to recruitment patterns, a feature of poly-substance users, or a function of the context. Furthermore, prospective studies would be well placed to conduct formative research to explore what would motivate and de-motivate poly-substance users from higher socio-economic backgrounds from participating in such surveys [28, 35].

High prevalence rates for methamphetamine use were evidenced in this study. This trend has also been noticed in increased admissions to specialist substance abuse treatment centres [36] suggesting that methamphetamine remains the more dominant substance of abuse in the metropole. Studies have shown that people using this drug are significantly more likely to report severe health, family and financial problems compared to people who used other substances [37]. Heroin and mandrax were also reported as commonly used and easily available beyond, of course, alcohol, tobacco and cannabis. There were slight variations across sites, for instance heroin seemed a more popular choice of drug for site 2 with at least 38 % of participants indicating heroin use. This could be explained by the fact that site 2 is a higher socio-economic area (with more disposable income when compared to the other two sites), is frequented by tourists, and therefore there are considerably more opportunities to earn an income through petty crime, begging or piecemeal work. This increases participants’ opportunity to engage in activities through which to source money, both in terms of frequency and in terms of the amounts they may be able to earn and what is needed to sustain their heroin use. Despite the bigger portion of the sample indicating that they did not inject heroin, non-injecting heroin users are at an increased risk for contracting HIV and other blood-borne diseases since risky sexual practices are associated with substance abuse [38, 39]. In addition, those who do not currently inject drugs may progress to injecting drug use as dependency develops and may therefore seek a more efficient means of administering their drug of choice [38]. These findings have serious implications for the delivery of prevention and treatment services, because if left untreated and unaddressed, it is likely that people using these substances will place a considerable burden on an already taxed health system in the province [37]. The findings highlight the need for evidence-based community programmes and other outreach activities aimed at recruiting substance users into specialised and structured treatment programmes.

We found high rates of poly-substance use and CAGE scores above the cut-off score (>2) with large numbers of people in all three sites reporting the use of three or more substances within the few days prior to the urine analysis. Poly-substance use potentially increases the likelihood of harm since illicit drugs that are taken concurrently or in close succession interact negatively in terms of their pharmacokinetic and pharmacodynamic mechanisms and can lead to the formation of unique psychoactive metabolites that may produce different effects than the original drug used [5, 40]. Additionally, studies conducted in Brazil showed that poly-substance users were at risk for interpersonal violence and thus more likely to be admitted to emergency rooms and more prone to cardiovascular and other disease [41]. Poly-substance use also increases the risk for both non- communicable and communicable diseases through increased exposure to HIV due to inconsistent condom use [42] and transactional sex. Longitudinal research investigating psychiatric trajectories among patients with poly-substance use also confirm that the use of multiple drugs and the presence of mental distress are strongly co-related over time [43, 44]. Given the high number of participants using three or more substances in this study, and the incumbent health and social burden associated with poly-substance use, the need for more comprehensive, integrated public health interventions that highlight not only single drug interventions but also the poly-substance perspective [43] is required.

The study also found that poly-substance users run the risk for arrest by virtue of either their use of illicit drugs or through selling or possession of illicit drugs. Conclusions drawn from a 3 metro study conducted in 2000 in three cities in South Africa however still hold true. Clear strategies to reduce drug use and drug related crime such as ensuring police are trained to facilitate diversions to treatment are needed [3].

Furthermore, our results indicate that very few poly-substance users in the three sites had ever accessed treatment, yet the majority reported the desire for treatment. Findings from a study in poorer disadvantaged communities in Cape Town found that people experience multiple barriers to accessing treatment that impacted on treatment need and thus service utilization [45]. Myers and colleagues suggest expanding existing treatment services to include low threshold services that target individuals with less severe problems [45]. In this respect, RDS recruitment strategies could be used to deliver tailored low threshold early intervention services or referral to specialist treatment centres for problematic poly-substance use given the difficulty of non-need barriers to treatment utilisation [45].

While our findings provide some insight into the effectiveness of using RDS in three diverse areas in the Western Cape, findings should be interpreted in the light of some limitations. First, given that this was a pilot study to test whether RDS is a methodology that could be used to recruit poly-substance users in three economically and social disparate sites only 100 participants were recruited from each site. Consequently, while RDS methods were applied, the study may fall short of full RDS assumptions, for example since the study it has been documented that the RDS-1 and RDS-2 estimators can suffer from serious bias, since it was a pilot. Second, recruitment ceased when 100 participants had been recruited and we did not take into account the number of eligible subjects each potential recruiter could have recruited beyond the pilot study (or the 100 participants). Third, our sample was recruited from a population of poly- substance users residing in three communities in Cape Town and cannot be generalized to other populations of substance users in South Africa.

## Conclusion

Our findings suggest a number of implications for RDS in the future and among other poly-substance-using populations. First, future studies can use RDS successfully to recruit poly-substance users. Second, given this preliminary success, RDS has the potential for successful use among poly-substance users for surveillance and monitoring over time. Third, the sampling strategy could be extended to recruitment of poly-substance users into prevention and early-interventions and facilitate referrals to specialist treatment centres. Finally, future research or recruitment of poly substance users should take into account the possible disparities in recruitment of women, and people of different ages and take measures to mitigate these potential biases.

## Abbreviations

CAGE: Cutting down, annoyance, guilt feelings, eye-opener
FAS: Family affluence scale
FGD: Focus group discussions
HIV: Human immunodeficiency virus
NRF: National research foundation (NRF
RDS: Respondent driven sampling
RDSAT: Respondent-driven sampling analysis tool 7.1.38
SACENDU: South African community epidemiology network of drug use
SES: Socio economic status
US$: American dollar (currency)
